# Association between red blood cell distribution width and white matter hyperintensities: A large‐scale cross‐sectional study

**DOI:** 10.1002/brb3.1739

**Published:** 2020-07-18

**Authors:** Meiyao Wang, Hongliang Feng, Shuaimei Zhang, Zhengjin Luo, Yan Liang, Yan Xu, Bin Mei, Zhaohong Kong, Yumin Liu

**Affiliations:** ^1^ Department of Neurology Zhongnan Hospital of Wuhan University Wuhan China; ^2^ Department of Ultrasonography Zhongnan Hospital of Wuhan University Wuhan China; ^3^ Department of Psychiatry Faculty of Medicine The Chinese University of Hong Kong Shatin, N.T. China; ^4^ Department of Neurology The First Affiliated Hospital Jinan University Guangzhou China; ^5^ Department of Neurology Renmin Hospital of Wuhan University Wuhan China

**Keywords:** cross‐sectional study, red blood cell distribution width, risk factors, white matter hyperintensities

## Abstract

**Background:**

Red blood cell distribution width (RDW) is a strong prognostic marker for various medical conditions, such as ischemic strokes. However, the relationships between higher RDW and the subtypes of white matter hyperintensities (WMHs) remain unclear. Hence, this study aimed to thoroughly evaluate the relationships between RDW and the subtypes of WMHs.

**Patients and methods:**

This cross‐sectional study was a retrospective analysis of hospital database (Dongguan Medical System, from April 2015 to February 2017). The presence and subtypes of WMHs were evaluated using Fazekas score with the T2WI‐FLAIR brain images from a 1.5‐T MRI system. The overall sample was randomly split in half. One of the two split‐half samples was used for determining the optimal cutoff value of higher RDW and another for further statistical analyses.

**Results:**

A total of 555 subjects with WMHs and 642 controls were recruited. The optimal cutoff value of higher RDW was 13.25%. Logistic regression revealed that higher RDW (≥13.25%) was positively associated with periventricular WMHs (adjusted OR = 1.81, 95% CI: 1.16–2.82, *p* = .009). However, higher RDW was not associated with total WMHs (adjusted OR = 1.52, 95% CI: 0.99–2.33, *p* = .057) and deep WMHs (adjusted OR = 1.21, 95% CI: 0.76–1.94, *p* = .426).

**Conclusion:**

Our findings suggested that higher RDW may be independently associated with periventricular WMHs, but not with total WMHs and deep WMHs.

## Graphical abstract

With the adequate sample size and comprehensive statistical analyses, our findings should be reliable and stable, which mainly suggest that higher RDW may be independently associated with the risk of WMHs and the risk of periventricular WMHs, but not with the deep WMHs.

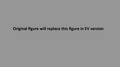

## 1 INTRODUCTION

The red blood cell distribution width (RDW) is routinely reported as part of the complete blood count, which represents the coefficient of variation for circulating red blood cell size (Hou et al., 2017). For a long time, RDW has been utilized in differentiating the causes of anemia in the clinical routine (Arbel et al., 2014; Novak, 1987). In addition, as an easy‐to‐measure marker for the systemic inflammatory response, RDW is widely used in many pathophysiological conditions, such as large B‐cell lymphoma and chronic lymphocytic leukemia (van Kimmenade et al., 2010; Loprinzi & Ford, 2015; Podhorecka et al., 2016; Yesil et al., 2011; Zhou et al., 2017). In recent years, there is an increasing body of evidence suggesting that RDW might be a useful indicator for cardiovascular morbidity, and all‐cause mortality and long‐term outcomes in patients with acute cerebral infarction (Arbel et al., 2014; Hou et al., 2017; Kim et al., 2012; van Kimmenade et al., 2010), and that RDW may serve as a novel predictor of dementia (Weuve, Mendes de Leon, Bennett, Dong, & Evans, 2014).

White matter hyperintensities (WMHs), one of the markers of cerebral small vessel disease, is commonly found in the periventricular and deep areas in brain imaging of the elderly, with the prevalence rate ranging from 50% to 95.6% (Longstreth et al., 1996; Moran, Phan, & Srikanth, 2012). Due to the growing population aging and the advances in neuroimaging technology, the detection rate of WMHs has been largely improved (Price et al., 2012). In 1987, Hachinski, Potter, and Merskey (1987) used the term “leukoaraiosis,” suggesting that these lesions could result in reduced x‐ray absorption in white matter. WMHs are mainly characterized by hyperintensity on T2‐weighted (T2WI) or fluid‐attenuated inversion‐recovery (FLAIR) magnetic resonance imaging (MRI). Compared to those without WMHs, subjects with WMHs had higher risks of experiencing ischemic strokes, gait disturbance, depression, dementia, incontinentia urinae, and even death, depending on the severity and lesion locations of WMHs (Wardlaw, Smith, & Dichgans, 2013; Zhong et al., 2016). Based on the distance from the lateral ventricles and correlations, WMHs can be divided into two subtypes: periventricular WMHs and deep WMHs (DeCarli, Fletcher, Ramey, Harvey, & Jagust, 2005).

To date, though growing evidence suggests that RDW may serve as a predictor of ischemic strokes, the association between RDW and WMHs is rarely investigated. By comparing 716 controls (Fazekas scale: 0–1) with 290 severe WMHs cases (Fazekas scale: 2–3), Lee et al. (2016) reported that higher RDW (≥13.3%) was independently related to the severity of WMHs. In addition, an independent correlation between higher RDW and WMHs was observed in the Chinese population with acute ischemic stroke (Peng et al.2017). However, given the potentially biased setting of the control group (Fazekas scale: 0–1) (Lee et al., 2016) or limited sample size (*n* = 125) (Peng et al.2017) of the previous studies, the association between RDW and WMHs has not been fully understood. Moreover, to the best of our knowledge, the relationships of higher RDW with the subtypes of WMHs (i.e., periventricular and deep WMHs) have never been investigated.

Therefore, this present study aimed to investigate the relationships of higher RDW with the subtypes of WMHs (total, periventricular, and deep WMHs).

## 2 PATIENTS AND METHODS

### 2.1 Study design and participant recruitment

This cross‐sectional study recruited subjects who visited the inpatient clinics of the Department of Neurology at Zhongnan Hospital of Wuhan University from April 2015 to February 2017. The study was approved by the Clinical Research Ethics Committee of Zhongnan Hospital of Wuhan University (Ref. No.: 2020097K). Since identifiable information would not be revealed and this study was based on a retrospective analysis of health care sources, the requirement for written consent forms from subjects was waived by the Research Ethics Committee.

Subjects were recruited if they fulfilled all of the following inclusion criteria: (a) their age at entry should range from 40 to 89 years old; (b) T2WI‐FLAIR brain images were available; and (c) demographical characteristics, laboratory results, and other related clinical data were obtainable. Subjects would be excluded if they met one of the following exclusion criteria: (a) having poor quality of T2WI‐FLAIR brain images; and (b) having a diagnosis of nonvascular central nervous system demyelinating diseases, such as multiple sclerosis, or central nervous system infections or intracranial tumors or craniocerebral trauma or hydrocephalus or other medical conditions that may affect the Fazekas score evaluation.

### 2.2 WMHs assessment

The T2WI‐FLAIR brain images of the subjects were from a 1.5‐T MRI system (Avanto; Erlangen Siemens). The WMHs assessment with the T2WI‐FLAIR brain images was performed by two neurologists who had sufficient training and experience in Fazekas scoring system (Fazekas, Chawluk, Alavi, Hurtig, & Zimmerman, 1987; Helenius, Goddeau, Moonis, & Henninger, 2016): (a) periventricular Fazekas scoring system: “0 = absent; 1 = caps or pencil lining; 2 = smooth halo; 3 = irregular periventricular hyperintensities extending into deep white matter”; and (b) deep Fazekas scoring system: “0 = absent; 1 = punctuate foci; 2 = beginning confluence of foci; 3 = large confluent areas.” According to the periventricular and deep Fazekas scores, subjects were divided into four groups: (a) control group: periventricular Fazekas score = 0 and deep Fazekas score = 0; (b) WMHs group: periventricular Fazekas score ≥ 1 or deep Fazekas score ≥ 1; (c) periventricular WMHs group: periventricular Fazekas score ≥ 1; and (d) deep WMHs group: deep Fazekas score ≥ 1. The inter‐rater reliability of the Fazekas scoring was substantial (*κ*: 0.63).

### 2.3 Assessment of RDW and other laboratory parameters

Red blood cell distribution width measurement was conducted with peripheral venous blood using an automated hematology analyzer Coulter STKS (Beckman Coulter). The Coulter STKS presented good performance with low false‐negative rate (1.8%), high sensitivity (96.3%), and specificity (83.3%) (Verheul, Spitters, & Bergmans, 1993). Other collected laboratory parameters included white blood cell count, red blood cell count, hemoglobin, platelet count, hematocrit, mean corpuscular volume, mean corpuscular hemoglobin, mean corpuscular hemoglobin concentration, fasting blood glucose, blood urea nitrogen, creatinine, uric acid, total cholesterol, triglyceride, high‐density lipoprotein cholesterol, and low‐density lipoprotein cholesterol.

### 2.4 Demographic and clinical characteristics

Demographic (i.e., age and sex) and clinical characteristics, including the diagnoses of cerebral artery atherosclerosis, hypertension, type 2 diabetes mellitus (T2DM), coronary artery disease (CAD), and anemia, were collected from the Dongguan Medical System.

### 2.5 Sample size estimation

The sample size estimation was based on the principle of 10 events per variable (EPV) for logistic regression (Harrell, 2015). As shown in Table 3, up to 13 variables were finally included in the multivariate logistic regression analyses. Therefore, at least 130 subjects with total WMHs, 130 subjects with periventricular WMHs and 130 subjects with deep WMHs were needed in this present study.

### 2.6 Statistical analysis

To avoid circular reasoning, half of the overall sample was randomly selected for the constructing receiver operating characteristic (ROC) curve. The optimal cutoff of higher RDW with the highest Youden's index was identified in ROC analysis based on the total WMHs and control groups. The remaining sample was used for subsequent analyses.

Categorical variables were presented as number (%) and continuous data were expressed as mean ± standard deviation. The inter‐group differences between control and any WMHs groups (total, periventricular and deep WMHs) were tested by *t* test or Kruskal–Wallis test or chi‐square test *t* test where applicable. Potential collinearity was estimated by Pearson correlation coefficients. If the correlation coefficients were greater than 0.8, collinearity was considered present (Holm, Carroll, Cassidy, Skillgate, & Ahlbom, 2007). Both univariate and multivariate logistic regressions were utilized to evaluate the associations between higher RDW and WMHs. In univariate and multivariate logistic regressions, the per quartiles of continuous variables were used. All statistical analyses were performed by SPSS v24.0. A value of two‐tailed *p* < .05 was considered statistically significant.

## 3 RESULTS

### 3.1 Clinical characteristics of subjects

Finally, a total of 1,197 subjects, including 555 subjects with total WMHs and 642 controls, were recruited. Among the overall sample, 277 subjects with total WMHs (49.9%) and 321 controls (50.0%) were randomly selected for constructing ROC. With the highest Youden's index, the optimal cutoff value of 13.25% for higher RDW was identified, which was closed to 13.0% determined by the previous study (Lee et al., 2016). The remaining 278 subjects with total WMHs (50.1%) and 321 controls (50.0%) were used for further statistical analyses. Among the 278 subjects with total WMHs of our study, 255 (91.7%) and 207 (74.5%) of them were classified into periventricular and deep WMHs groups, respectively.

The demographic, clinical, and laboratory characteristics among the groups are shown in Table 1. Subjects with total WMHs (61.9% vs. 48.3%, *p* < .05), subjects with periventricular WMHs (65.1% vs. 48.3%, *p* < .05), and subjects with deep WMHs (59.4% vs. 48.3%, *p* < .05) presented higher rates of higher RDW than controls. Compared with control group, total, periventricular, and deep WMHs groups also had significant older age, higher rates of cerebral artery atherosclerosis, hypertension, T2DM, anemia, and higher levels of blood urea nitrogen, creatinine, and uric acid (all *p* < .05). In addition, total WMHs, periventricular WMHs, and deep WMHs groups showed significantly lower levels of red blood cell count, hemoglobin, hematocrit, and low‐density lipoprotein cholesterol (all *p* < .05). There was no significant difference in sex (male), white blood cell count, platelet count, mean corpuscular volume, mean corpuscular hemoglobin, fasting blood glucose, triglyceride, and high‐density lipoprotein cholesterol between any WMHs group and control group (all *p* > .05).

**Table 1:** Demographic, clinical, and laboratory characteristics of subjects

	Controls (*n* = 321)	Subjects with WMHs (*n* = 278)	Subjects with periventricular WMHs (*n* = 255)	Subjects with deep WMHs (*n* = 207)
Higher RDW (≥13.25%)	155 (48.3)	**172 (61.9)**	**166 (65.1)**	**123 (59.4)**
Age (year)	59.5 ± 9.9	**70.4 ± 9.8**	**71.0 ± 9.6**	**71.0 ± 9.7**
Male (%)	150 (46.7)	126 (45.3)	116 (45.5)	96 (46.4)
Cerebral artery atherosclerosis (%)	49 (15.2)	**81 (29.2)**	**77 (30.2)**	**54 (30.2)**
Hypertension (%)	125 (38.9)	**204 (73.4）**	**189 (74.1)**	**157 (75.8)**
Diabetes mellitus type 2 (%)	46 (14.3)	**61 (21.9）**	**59 (23.1)**	**46 (22.2)**
Coronary artery disease (%)	19 (5.9)	24 (8.6)	**24 (9.4)**	15 (7.2)
Anemia	30 (9.3)	**45 (16.2)**	**42 (16.5)**	**34 (16.4)**
White blood cell count (10^9^/L)	6.5 ± 2.3	6.3 ± 2.0	6.5 ± 2.3	6.2 ± 2.0
Red blood cell count (10^12^/L)	4.4 ± 0.5	**4.2 ± 0.5**	**4.2 ± 0.5**	**4.2 ± 0.5**
Hemoglobin (G/L)	131.6 ± 14.6	**127.6 ± 15.3**	**128.0 ± 15.4**	**127.3 ± 15.7**
Platelet count (10^9^/L)	193.8 ± 55.9	189.1 ± 57.8	189.0 ± 58.4	185.7 ± 57.9
Hematocrit (%)	39.9 ± 4.3	**38.8 ± 4.5**	**38.9 ± 4.6**	**38.7 ± 4.7**
Mean corpuscular volume (fL)	91.9 ± 6.2	92.4 ± 5.5	92.5 ± 5.6	92.2 ± 6.0
Mean corpuscular hemoglobin (pg)	30.3 ± 2.3	30.4 ± 2.0	30.4 ± 2.0	30.3 ± 2.1
Mean corpuscular hemoglobin concentration (g/L)	329.5 ± 6.7	329.0 ± 6.2	**328.9 ± 6.2**	**328.7 ± 6.3**
Fasting blood glucose (mM)	5.7 ± 2.1	5.9 ± 2.0	5.2 ± 1.7	6.0 ± 2.1
Blood urea nitrogen (mM)	5.0 ± 1.3	**5.1 ± 1.6**	**5.4 ± 1.9**	**5.3 ± 1.6**
Creatinine (μM)	67.2 ± 17.5	**74.0 ± 22.8**	**74.4 ± 23.2**	**74.2 ± 20.8**
Uric Acid (μM)	309.1 ± 93.7	**326.4 ± 101.1**	**325.4 ± 99.3**	**335.3 ± 105.5**
Total Cholesterol (mM)	4.6 ± 0.9	4.5 ± 1.0	**4.6 ± 1.0**	**4.4 ± 0.9**
Triglyceride (mM)	1.7 ± 1.2	1.6 ± 1.0	1.7 ± 1.0	1.6 ± 0.9
High‐density lipoprotein cholesterol (mM)	1.3 ± 0.3	1.3 ± 0.4	1.3 ± 0.3	1.3 ± 0.4
Low‐density lipoprotein cholesterol (mM)	2.8 ± 0.8	**2.7 ± 0.8**	**2.7 ± 0.9**	**2.7 ± 0.8**

Note

Data are shown as mean ± standard deviation or *n* (%).Statistically significant differences (*p*‐value < .05) by *t* test or chi‐square test, compared with control group respectively, are highlighted in bold.

Abbreviations: RDW, red blood cell distribution width; WMHs, white matter hyperintensities.

### 3.2 Univariate logistic regression

As shown in Table 2, higher RDW were positively associated with total WMHs (OR = 1.74, 95% CI = 1.25–2.41, *p* < .001), periventricular WMHs (OR = 2.00, 95% CI = 1.43–2.80, *p* < .001), and deep WMHs (OR = 1.57, 95% CI = 1.10–2.23, *p* = .013). There were also significant relationships of WMHs (total, periventricular, and deep WMHs) with age, cerebral artery atherosclerosis, hypertension, T2DM, anemia, red blood cell count, hemoglobin, creatinine, and low‐density lipoprotein cholesterol (all *p* < .05). In addition, any WMHs (total, periventricular, and deep WMHs) was not related to sex (male), CAD, white blood cell count, mean corpuscular volume, mean corpuscular hemoglobin, mean corpuscular hemoglobin concentration, triglyceride, and high‐density lipoprotein cholesterol (all *p* > .05).

**Table 2:** Results of univariate logistic regression for the different types of WMHs

	Subjects with WMHs (*n* = 278)		Subjects with periventricular WMHs (*n* = 255)		Subjects with deep WMHs (*n* = 207)	
	OR [95% CI]	*p*	OR [95% CI]	*p*	OR [95% CI]	*p*
Higher RDW (≥ 13.25%)	**1.74 [1.25–2.41]**	**<.001**	**2.00 [1.43–2.80]**	**<.001**	**1.57 [1.10–2.23]**	**.013**
Age (per 10 years)	**2.59 [2.15–3.12]**	**<.001**	**2.74 [2.25–3.32]**	**<.001**	**2.66 [2.17–3.25]**	**<.001**
Male	0.95 [0.69–1.30]	.731	0.95 [0.68–1.32]	.767	0.99 [0.70–1.40]	.937
Cerebral artery atherosclerosis	**2.30 [1.50–3.53]**	**<.001**	**2.41 [1.56–3.73]**	**<.001**	**2.41 [1.52–3.81]**	**<.001**
Hypertension	**4.32 [3.05–6.12]**	**<.001**	**4.49 [3.14–6.43]**	**<.001**	**4.92 [3.34–7.27]**	**<.001**
Diabetes mellitus type 2	**1.68 [1.10–2.56]**	**.016**	**1.80 [1.17–2.76]**	**.007**	**1.71 [1.09–2.69]**	**.020**
Coronary artery disease	1.50 [0.80–2.81]	.202	1.65 [0.88–3.09]	.116	1.24 [0.62–2.50]	.545
Anemia	**1.87 [1.14–3.07]**	**.013**	**1.91 [1.16–3.16]**	**.011**	**2.91 [1.13–3.23]**	**.016**
White blood cell count (per quartile)	1.01 [0.88–1.17]	.851	1.04 [0.89–1.21]	.628	1.00 [0.85–1.17]	.994
Red blood cell count (per quartile)	**0.75 [0.64–0.87]**	**<.001**	**0.75 [0.65–0.88]**	**<.001**	**0.74 [0.63–0.87]**	**<.001**
Platelet count (per quartile)	0.89 [0.77–1.03]	.124	0.89 [0.77–1.03]	.123	**0.84 [0.72–0.99]**	**.033**
Hematocrit (per quartile)	**0.77 [0.67–0.90]**	**.001**	**0.79 [0.68–0.92]**	**.002**	**0.75 [0.64–0.89]**	**.001**
Mean corpuscular volume (per quartile)	1.01 [0.87–1.16]	.922	1.01 [0.88–1.18]	.850	0.97 [0.83–1.14]	.725
Mean corpuscular hemoglobin (per quartile)	0.96 [0.83–1.10]	.531	0.96 [0.83–1.11]	.537	0.91 [0.78–1.06]	.238
Mean corpuscular hemoglobin concentration (per quartile)	0.91 [0.79–1.06]	.241	0.90 [0.77–1.05]	.192	0.89 [0.76–1.05]	.168
Fasting blood‐glucose (per quartile)	**1.16 [1.00–1.34]**	**.044**	**1.17 [1.01–1.36]**	.034	1.14 [0.98–1.33]	.093
Blood urea nitrogen (per quartile)	1.11 [0.96–1.28]	.157	1.07 [0.92–1.23]	.399	**1.18 [1.01–1.38]**	**.036**
Creatinine (per quartile)	**1.38 [1.19–1.60]**	**<.001**	**1.40 [1.21–1.63]**	**<.001**	**1.40 [1.20–1.65]**	**<.001**
Uric acid (per quartile)	1.15 [1.00–1.33]	.051	1.15 [1.00–1.33]	.054	**1.24 [1.06–1.45]**	**.007**
Total cholesterol (per quartile)	0.89 [0.77–1.02]	.094	0.88 [0.77–1.02]	.088	**0.84 [0.72–0.98]**	**.029**
Triglyceride (per quartile)	0.99 [0.85–1.14]	.837	1.01 [0.87–1.17]	.918	0.96 [0.82–1.12]	.574
High‐density lipoprotein cholesterol (per quartile)	0.93 [0.80–1.07]	.285	0.90 [0.78–1.04]	.165	0.90 [0.77–1.05]	.163
Low‐density lipoprotein cholesterol (per quartile)	**0.85 [0.74–0.98]**	**.026**	**0.84 [0.72–0.97]**	**.018**	**0.82 [0.70–0.96]**	**.012**

Note

Statistically significant differences (*p*‐value < .05) are highlighted in bold.

Abbreviations: 95% CI, 95% confidence interval; OR, odds ratio; RDW, red blood cell distribution width; WMHs, white matter hyperintensities.

### 3.3 Multicollinearity evaluation

The matrices of Pearson correlation coefficients (Tables S1–S3: online resource) show the associations among the demographic, clinical, and laboratory parameters. The red blood cell count, hemoglobin, and hematocrit were highly correlated with each other (*r* ≥.8). The mean corpuscular volume was highly correlated with mean corpuscular hemoglobin (*r* > .8). In addition, total cholesterol was highly associated with low‐density lipoprotein cholesterol (*r* > .8). Therefore, if more than one variables from certain cluster were found significantly correlated with (total, periventricular, and deep) WMHs in univariate logistic regression (Table 2), only one of them would be included in the multivariate logistic regression.

### 3.4 Multivariate logistic regression

After controlling for age, cerebral artery atherosclerosis, hypertension, T2DM, anemia, red blood cell count, fasting blood glucose, and low‐density lipoprotein cholesterol, higher RDW was positively associated with periventricular WMHs (adjusted OR = 1.81, 95% CI: 1.16–2.82, *p* = .009), but not with total WMHs (adjusted OR = 1.52, 95% CI: 0.99–2.33, *p* = .057) (Table 3). In addition, there was no significant association between higher RDW and deep WMHs (adjusted OR = 1.21, 95% CI: 0.76–1.94, *p* = .426), after controlling for age, cerebral artery atherosclerosis, hypertension, T2DM, anemia, red blood cell count, platelet count, fasting blood glucose, blood urea nitrogen, uric acid, and low‐density lipoprotein cholesterol.

**Table 3:** Results of multivariate logistic regression for the different types of WMHs

	Subjects with WMHs (*n* = 278)		Subjects with periventricular WMHs (*n* = 255)		Subjects with deep WMHs (*n* = 207)	
	OR [95% CI]	*p*	OR [95% CI]	*p*	OR [95% CI]	*p*
Higher RDW (≥13.25%)	1.52 [0.99–2.33]	.057	**1.81 [1.16–2.82]**	**.009**	1.21 [0.76–1.94]	.426
Age (per 10 years)	**2.28 [1.82–2.86]**	**<.001**	**2.41 [1.90–3.06]**	**<.001**	**2.24 [1.75–2.88]**	**<.001**
Cerebral artery atherosclerosis	1.37 [0.83–2.27]	.220	1.36 [0.81–2.29]	.240	1.42 [0.83–2.44]	.206
Hypertension	**3.31 [2.15–5.09]**	**<.001**	**3.21 [2.05–5.01]**	**<.001**	**3.64 [2.23–5.94]**	**<.001**
Diabetes mellitus type 2	1.01 [0.56–1.84]	.971	1.14 [0.62–2.10]	.681	0.90 [0.47–1.74]	.761
Anemia	0.84 [0.42–1.66]	.614	0.82 [0.41–1.66]	.582	0.88 [0.42–1.83]	.723
Red blood cell count (per quartile)	0.82 [0.66–1.02]	.075	0.86 [0.68–1.08]	.186	0.85 [0.67–1.08]	.179
Platelet count (per quartile)	—	—	—	—	0.94 [0.76–1.16]	.553
Fasting blood‐glucose (per quartile)	**1.26 [1.02–1.56]**	**.031**	1.26 [1.01–1.57]	.039	**1.28 [1.02–1.62]**	**.034**
Blood urea nitrogen (per quartile)	—	—	—	—	1.01 [0.81–1.27]	.900
Creatinine (per quartile)	1.14 [0.93–1.38]	.203	**1.15 [0.94–1.41]**	.172	1.05 [0.82–1.35]	.678
Uric acid (per quartile)	—	—	—	—	1.07 [0.85–1.35]	.549
Low‐density lipoprotein cholesterol (per quartile)	1.02 [0.84–1.23]	.862	1.00 [0.82–1.21]	.980	0.98 [0.79–1.21]	.839

Note

Statistically significant differences (*p‐*value < .05) are highlighted in bold.

Abbreviations: 95% CI, 95% confidence interval; OR, odds ratio; RDW, red blood cell distribution width; WMHs, white matter hyperintensities.

## 4 DISCUSSION

To our best knowledge, this study provided the first evidence investigating the relationships of higher RDW with different subtypes of WMHs. The significant relationships of higher RDW with total, periventricular, and deep WMHs were identified in the univariate logistic regressions. To further investigate these associations, multivariate logistic regressions were conducted. The multivariate logistic regression demonstrated that higher RDW were still positively correlated with periventricular WMHs (*p* = .009), even after controlling confounding factors. However, the significant relationships of higher RDW with total WMHs (*p* = .057) and deep WMHs disappeared (*p* = .426) after controlling confounding factors.

Lee et al. (2016) reported that higher RDW was independently correlated with the severity of WMHs when compared severe WMHs cases (Fazekas scale: 2–3) with absent to mild WMHs cases (Fazekas scale: 0–1). Similarly, a significant correlation between RDW and the severity of WMHs was observed in the Chinese population with acute ischemic stroke (Peng et al.2017). To some extent, our findings were not totally consistent with these previous studies. Among the 278 subjects with total WMHs of our study, 255 (91.7%) and 207 (74.5%) of them were classified into periventricular and deep WMHs groups, respectively. Therefore, the association between higher RDW and total WMHs may vary depending on the presence of periventricular and deep WMHs.

Hypoperfusion may be one explanation for the correlation between higher RDW and periventricular WMHs. It is well known that hemispheric white matter is mainly dominated by deep perforating arteries, most of which are terminal branches without much collateral circulation (Du, Keyoung, Dowd, Young, & Lawton, 2007; Stoeckel, Wittsack, Meisel, & Seitz, 2007). In addition, the periventricular white matter is located in border zones, where brain tissue is particularly susceptible to ischemic damage when blood flow changes abnormally (Mandell et al., 2008; Novak et al., 2006; Pantoni & Garcia, 1997). In this regard, chronic ischemia/hypoperfusion is thought to be the most important pathophysiological mechanism of WMHs. To date, this hypothesis has been well approved by many functional neuroimaging studies (Sanossian et al., 2011). On the other hand, it has been shown that elevated RDW was strongly associated with the loss of red blood cell deformability (Patel et al., 2013). Red blood cells with impaired deformability may be unable to squeeze through the capillaries, thus impairing blood flow through the microcirculation or blocking the small vessels (Ballas & Smith, 1992). Thus, higher RDW may directly contribute to the onset of periventricular WMHs through undermining the perfusion of periventricular regions.

In addition, both higher RDW and periventricular WMHs may share the same pathophysiological basis—inflammation. On the one hand, the Northern Manhattan Study (Wright et al., 2009) demonstrated that the inflammatory markers, including lipoprotein‐associated phospholipase A2 (Lp‐PLA2) and myeloperoxidase, were correlated with a greater burden of WMHs. On the other hand, the higher level of RDW may be an indicator of inflammation in the human body, since inflammation may lead to anisocytosis through causing ineffective erythropoiesis (Arbel et al., 2014; Simel, DeLong, Feussner, Weinberg, & Crawford, 1988). Collectively, the elevated RDW may serve as a novel biomarker for the onset of WMHs. In addition, to enhance our better understanding of the pathogenesis of WMHs, future studies should pay attention to inflammatory markers when investigating the relationship between RDW and WMHs.

This study has some strengths to be worthy of noting. First, to avoid circular reasoning, the overall sample was randomly split in half. One of the two split‐half samples was used for identifying the optimal cutoff value of higher RDW and another for further statistical analyses. Second, to thoroughly explore the association between higher RDW and WMHs, both the presence and subtypes of WMHs were evaluated. However, the limitations of this study should be noted. First, as the subjects of this study were recruited from inpatient clinics, the findings may not be well generalized to the general population. Second, other potential confounding factors, such as inflammatory markers and lifestyle (e.g., smoking, diet, physical activity) (Espeland et al., 2016; Power et al., 2015), were not included in this study. Third, the cross‐sectional design of this study did not allow us to investigate causal relationships.

## 5 CONCLUSION

To conclude, higher RDW is independently associated with periventricular WMHs but not with deep WMHs. Further prospective studies with a large sample size are warranted to verify these associations between higher RDW and the subtypes of WMHs.

## CONFLICT OF INTEREST

The authors declare that there are no conflicts of interest.

## AUTHOR CONTRIBUTIONS

Meiyao Wang, Hongliang Feng, and Yumin Liu conceived and designed the trial; Shuaimei Zhang and Zhengjin Luo collected the data; Meiyao Wang, Hongliang Feng, and Yumin Liu analyzed the data and wrote this paper; Yan Liang, Yan Xu, Bin Mei, and Zhaohong Kong collected the data and revised the paper.

## Supporting information

Table S1‐S3

## Data Availability

The data that support the findings of this study are available on request from the corresponding author. The data are not publicly available due to privacy.
