# Supplementary material for: Association between red blood cell distribution width and white matter hyperintensities: A large‐scale cross‐sectional study
Source: Brain Behav. 2020 Jul 18;10(9):e01739. doi: 10.1002/brb3.1739 (PMC7503097; doi:10.1002/brb3.1739)
Supplement: Supplementary file 1 — Table S1‐S3 [file BRB3-10-e01739-s001.doc]

| **Supplementary Table 1. Matrix of** **Pearson correlation coefficients among independent variables for the relative factor analyses of WMHs** | Male | 1 |  |  |  |  |  |  |  |  |  |  |  |  |  |  |  |  |  |  |  |  |  |  | CAA: cerebral artery atherosclerosis; T2DM: diabetes mellitus type 2; CAD: coronary artery disease; WBC: white blood cell count; RBC: red blood cell count; HGB: hemoglobin; PLT: platelet count; CT: hematocrit; MCV: mean corpuscular volume; MCH: mean corpuscular hemoglobin; MCHC: mean corpuscular hemoglobin concentration; RDW: red blood cell distribution width; FBG: fasting blood-glucose; BUN: Blood Urea Nitrogen; CREA: creatinine; UA: uric acid; TC: total cholesterol; TG: triglyceride; HDL-C: high-density lipoprotein cholesterol; LDL-C: low-density lipoprotein cholesterol. Statistically significant differences (P-value < 0.05) are highlighted in bold. |
| --- | --- | --- | --- | --- | --- | --- | --- | --- | --- | --- | --- | --- | --- | --- | --- | --- | --- | --- | --- | --- | --- | --- | --- | --- | --- |
|  | Age | **-0.055** | 1 |  |  |  |  |  |  |  |  |  |  |  |  |  |  |  |  |  |  |  |  |  |  |
|  | CAA | 0.044 | **0.125** | 1 |  |  |  |  |  |  |  |  |  |  |  |  |  |  |  |  |  |  |  |  |  |
|  | Hypertension | 0.041 | **0.267** | **0.197** | 1 |  |  |  |  |  |  |  |  |  |  |  |  |  |  |  |  |  |  |  |  |
|  | 2-DM | -0.021 | **0.104** | **0.153** | **0.189** | 1 |  |  |  |  |  |  |  |  |  |  |  |  |  |  |  |  |  |  |  |
|  | CAD | -0.022 | **0.114** | 0.025 | **0.12** | **0.092** | 1 |  |  |  |  |  |  |  |  |  |  |  |  |  |  |  |  |  |  |
|  | Anemia | 0.025 | **0.249** | 0.003 | 0.049 | 0.008 | 0.032 | 1 |  |  |  |  |  |  |  |  |  |  |  |  |  |  |  |  |  |
|  | WBC | **0.156** | **-0.076** | **0.134** | **0.128** | **0.101** | 0.013 | **-0.12** | 1 |  |  |  |  |  |  |  |  |  |  |  |  |  |  |  |  |
|  | RBC | **0.372** | **-0.306** | -0.007 | -0.012 | 0.009 | -0.027 | **-0.414** | **0.262** | 1 |  |  |  |  |  |  |  |  |  |  |  |  |  |  |  |
|  | HGB | **0.491** | **-0.27** | -0.014 | -0.016 | -0.023 | -0.039 | **-0.547** | **0.252** | **0.8** | 1 |  |  |  |  |  |  |  |  |  |  |  |  |  |  |
|  | PLT | **-0.068** | **-0.161** | 0.044 | -0.002 | -0.044 | -0.025 | -0.041 | **0.271** | **0.113** | 0.04 | 1 |  |  |  |  |  |  |  |  |  |  |  |  |  |
|  | HCT | **0.489** | **-0.246** | -0.018 | -0.014 | -0.033 | -0.036 | **-0.54** | **0.262** | **0.825** | **0.945** | **0.063** | 1 |  |  |  |  |  |  |  |  |  |  |  |  |
|  | MCV | **0.229** | **0.104** | **-0.076** | -0.03 | **-0.107** | -0.004 | -0.080 | -0.023 | **-0.213** | **0.159** | **-0.117** | **0.155** | 1 |  |  |  |  |  |  |  |  |  |  |  |
|  | MCH | **0.243** | **0.059** | **-0.086** | -0.054 | **-0.078** | -0.02 | **-0.113** | -0.038 | **-0.175** | **0.209** | **-0.119** | **0.156** | **0.875** | 1 |  |  |  |  |  |  |  |  |  |  |
|  | MCHC | **0.062** | **-0.07** | -0.056 | -0.032 | **0.092** | -0.048 | **-0.176** | -0.048 | -0.018 | **0.182** | **-0.1** | **0.041** | **0.121** | **0.41** | 1 |  |  |  |  |  |  |  |  |  |
|  | FPG | 0.005 | **0.02** | **0.072** | **0.089** | **0.413** | 0.023 | **-0.084** | **0.171** | **0.118** | **0.113** | **0.071** | **0.087** | **-0.097** | -0.036 | **0.163** | 1 |  |  |  |  |  |  |  |  |
|  | BUN | **0.186** | **0.098** | **0.063** | **0.068** | **0.094** | 0.02 | **0.085** | **0.056** | -0.004 | 0.042 | **-0.061** | 0.039 | 0.035 | **0.013** | 0.019 | **0.085** | 1 |  |  |  |  |  |  |  |
|  | CREA | **0.536** | **0.153** | **0.088** | **0.182** | 0.014 | 0.008 | **0.131** | **0.072** | **0.159** | **0.246** | **-0.112** | **0.25** | **0.174** | 0.147 | -0.006 | -0.051 | **0.347** | 1 |  |  |  |  |  |  |
|  | UA | **0.334** | 0.029 | 0.046 | **0.22** | 0.031 | 0.021 | 0.001 | **0.13** | **0.178** | **0.194** | -0.032 | **0.195** | 0.029 | 0.034 | 0.017 | -0.002 | **0.163** | **0.421** | 1 |  |  |  |  |  |
|  | TC | **-0.129** | **-0.157** | 0.005 | **-0.079** | 0.008 | -0.058 | **-0.234** | **0.07** | **0.219** | **0.189** | **0.213** | **0.178** | -0.065 | -0.059 | 0.013 | **0.124** | 0.007 | **-0.08** | -0.019 | 1 |  |  |  |  |
|  | TG | -0.005 | **-0.103** | **0.077** | **0.153** | **0.215** | -0.028 | **-0.154** | **0.15** | **0.225** | **0.198** | **0.111** | **0.19** | **-0.095** | -0.028 | **0.133** | **0.159** | -0.038 | 0.041 | **0.243** | **0.274** | 1 |  |  |  |
|  | HDL-C | **-0.273** | 0.004 | **-0.137** | **-0.097** | **-0.14** | 0.019 | -0.078 | **-0.152** | **-0.104** | **-0.104** | 0.03 | **-0.107** | 0.029 | 0.008 | -0.056 | **-0.086** | 0.012 | **-0.172** | **-0.271** | **0.213** | **-0.368** | 1 |  |  |
|  | LDL-C-C | **-0.038** | **-0.16** | 0.012 | **-0.079** | -0.032 | -0.033 | **-0.216** | **0.****069** | **0.248** | **0.23** | **0.186** | **0.217** | -0.046 | -0.044 | 0.011 | **0.112** | -0.011 | -0.043 | -0.008 | **0.826** | **0.152** | **0.091** | 1 |  |
|  |  | Male | Age | CAA | Hypertension | T2DM | CAD | Anemia | WBC | RBC | HGB | PLT | HCT | MCV | MCH | MCHC | FPG | BUN | CREA | UA | TC | TG | HDL-C | LDL-C-C |  |

| **Supplementary Table 2. Matrix of Pearson correlation coefficients among independent variables for the relative factor analyses of periventricular WMHs** | Male | 1 |  |  |  |  |  |  |  |  |  |  |  |  |  |  |  |  |  |  |  |  |  |  | CAA: cerebral artery atherosclerosis; T2DM: diabetes mellitus type 2; CAD: coronary artery disease; WBC: white blood cell count; RBC: red blood cell count; HGB: hemoglobin; PLT: platelet count; CT: hematocrit; MCV: mean corpuscular volume; MCH: mean corpuscular hemoglobin; MCHC: mean corpuscular hemoglobin concentration; RDW: red blood cell distribution width; FBG: fasting blood-glucose; BUN: Blood Urea Nitrogen; CREA: creatinine; UA: uric acid; TC: total cholesterol; TG: triglyceride; HDL-C: high-density lipoprotein cholesterol; LDL-C: low-density lipoprotein cholesterol. Statistically significant differences (P-value < 0.05) are highlighted in bold. |
| --- | --- | --- | --- | --- | --- | --- | --- | --- | --- | --- | --- | --- | --- | --- | --- | --- | --- | --- | --- | --- | --- | --- | --- | --- | --- |
|  | Age | -0.055 | 1 |  |  |  |  |  |  |  |  |  |  |  |  |  |  |  |  |  |  |  |  |  |  |
|  | CAA | 0.04 | 0.072 | 1 |  |  |  |  |  |  |  |  |  |  |  |  |  |  |  |  |  |  |  |  |  |
|  | Hypertension | 0.061 | **0.203** | **0.194** | 1 |  |  |  |  |  |  |  |  |  |  |  |  |  |  |  |  |  |  |  |  |
|  | 2-DM | -0.025 | **0.116** | **0.206** | **0.205** | 1 |  |  |  |  |  |  |  |  |  |  |  |  |  |  |  |  |  |  |  |
|  | CAD | -0.03 | **0.167** | 0.011 | **0.146** | 0.08 | 1 |  |  |  |  |  |  |  |  |  |  |  |  |  |  |  |  |  |  |
|  | Anemia | 0.025 | **0.249** | 0.003 | 0.049 | 0.008 | 0.032 | 1 |  |  |  |  |  |  |  |  |  |  |  |  |  |  |  |  |  |
|  | WBC | **0.141** | **-0.124** | **0.153** | **0.123** | **0.093** | 0.032 | **-0.12** | 1 |  |  |  |  |  |  |  |  |  |  |  |  |  |  |  |  |
|  | RBC | **0.4** | **-0.287** | -0.001 | 0.009 | -0.016 | -0.032 | **-0.414** | **0.267** | 1 |  |  |  |  |  |  |  |  |  |  |  |  |  |  |  |
|  | HGB | **0.527** | **-0.252** | -0.044 | 0.022 | -0.048 | -0.03 | **-0.547** | **0.253** | **0.789** | 1 |  |  |  |  |  |  |  |  |  |  |  |  |  |  |
|  | PLT | -0.069 | **-0.148** | **0.098** | 0.037 | -0.058 | 0.005 | -0.041 | **0.265** | **0.108** | 0.029 | 1 |  |  |  |  |  |  |  |  |  |  |  |  |  |
|  | HCT | **0.528** | **-0.229** | -0.047 | 0.025 | -0.054 | -0.021 | **-0.54** | **0.263** | **0.827** | **0.944** | 0.053 | 1 |  |  |  |  |  |  |  |  |  |  |  |  |
|  | MCV | **0.258** | **0.126** | **-0.106** | -0.003 | **-0.087** | 0.027 | -0.080 | -0.027 | **-0.181** | **0.2** | **-0.152** | **0.186** | 1 |  |  |  |  |  |  |  |  |  |  |  |
|  | MCH | **0.27** | **0.107** | **-0.109** | -0.023 | -0.071 | 0.012 | **-0.113** | -0.048 | **-0.157** | **0.237** | **-0.158** | **0.178** | **0.868** | 1 |  |  |  |  |  |  |  |  |  |  |
|  | MCHC | **0.089** | -0.01 | -0.039 | 0.011 | 0.062 | -0.009 | **-0.176** | -0.072 | -0.03 | **0.186** | **-0.117** | 0.043 | **0.117** | **0.422** | 1 |  |  |  |  |  |  |  |  |  |
|  | FPG | 0.025 | -0.005 | **0.09** | 0.083 | **0.388** | -0.003 | **-0.084** | **0.161** | **0.112** | **0.109** | 0.082 | 0.081 | **-0.1** | -0.038 | **0.156** | 1 |  |  |  |  |  |  |  |  |
|  | BUN | **0.219** | 0.053 | 0.054 | 0.049 | **0.112** | -0.005 | **0.085** | 0.074 | 0.018 | 0.06 | -0.035 | 0.053 | 0.052 | 0.026 | 0.035 | **0.115** | 1 |  |  |  |  |  |  |  |
|  | CREA | **0.59** | **0.093** | 0.063 | **0.161** | 0.017 | -0.034 | **0.131** | 0.062 | **0.216** | **0.302** | **-0****.099** | **0.307** | **0.213** | **0.185** | 0.026 | -0.037 | **0.339** | 1 |  |  |  |  |  |  |
|  | UA | **0.373** | 0.021 | 0.022 | **0.224** | 0.055 | 0.01 | 0.001 | **0.131** | **0.235** | **0.249** | -0.041 | **0.258** | 0.049 | 0.048 | 0.011 | 0.023 | **0.157** | **0.41** | 1 |  |  |  |  |  |
|  | TC | **-0.129** | **-0.125** | -0.021 | -0.075 | 0.022 | -0.068 | **-0.234** | 0.091 | **0.204** | **0.166** | **0.205** | **0.157** | **-0.085** | **-0.097** | 0.001 | **0.162** | 0.028 | **-0.101** | -0.018 | 1 |  |  |  |  |
|  | TG | 0.043 | -0.052 | **0.093** | **0.202** | **0.209** | -0.038 | **-0.154** | **0.189** | **0.245** | **0.205** | **0.107** | **0.206** | **-0.089** | -0.033 | **0.091** | **0.142** | -0.042 | 0.042 | **0.258** | **0.239** | 1 |  |  |  |
|  | HDL-C | **-0.294** | 0.001 | **-0.117** | **-0.103** | **-0.117** | 0.027 | -0.078 | **-0.163** | **-0.102** | **-0.108** | 0.003 | **-0.116** | 0.017 | -0.007 | -0.034 | -0.079 | -0.002 | **-0.166** | **-0.289** | **0.201** | **-0.387** | 1 |  |  |
|  | LDL-C | -0.027 | **-0.****146** | -0.018 | **-0.088** | -0.027 | -0.055 | **-0.216** | 0.077 | **0.238** | **0.213** | **0.165** | **0.204** | -0.075 | -0.09 | -0.006 | **0.135** | 0.01 | -0.058 | -0.009 | **0.804** | **0.114** | 0.068 | 1 |  |
|  |  | Male | Age | CAA | Hypertension | T2DM | CAD | Anemia | WBC | RBC | HGB | PLT | HCT | MCV | MCH | MCHC | FPG | BUN | CREA | UA | TC | TG | HDL-C | LDL-C |  |

| **Supplementary Table 3. Matrix of Pearson correlation coefficients among independent variables for the relative factor analyses of deep WMHs** | Male | 1 |  |  |  |  |  |  |  |  |  |  |  |  |  |  |  |  |  |  |  |  |  |  | CAA: cerebral artery atherosclerosis; T2DM: diabetes mellitus type 2; CAD: coronary artery disease; WBC: white blood cell count; RBC: red blood cell count; HGB: hemoglobin; PLT: platelet count; CT: hematocrit; MCV: mean corpuscular volume; MCH: mean corpuscular hemoglobin; MCHC: mean corpuscular hemoglobin concentration; RDW: red blood cell distribution width; FBG: fasting blood-glucose; BUN: Blood Urea Nitrogen; CREA: creatinine; UA: uric acid; TC: total cholesterol; TG: triglyceride; HDL-C: high-density lipoprotein cholesterol; LDL-C: low-density lipoprotein cholesterol. Statistically significant differences (P-value < 0.05) are highlighted in bold. |
| --- | --- | --- | --- | --- | --- | --- | --- | --- | --- | --- | --- | --- | --- | --- | --- | --- | --- | --- | --- | --- | --- | --- | --- | --- | --- |
|  | Age | -0.062 | 1 |  |  |  |  |  |  |  |  |  |  |  |  |  |  |  |  |  |  |  |  |  |  |
|  | CAA | 0.072 | 0.076 | 1 |  |  |  |  |  |  |  |  |  |  |  |  |  |  |  |  |  |  |  |  |  |
|  | Hypertension | **0.085** | **0.159** | **0.146** | 1 |  |  |  |  |  |  |  |  |  |  |  |  |  |  |  |  |  |  |  |  |
|  | 2-DM | -0.005 | **0.126** | **0.183** | **0.206** | 1 |  |  |  |  |  |  |  |  |  |  |  |  |  |  |  |  |  |  |  |
|  | CAD | -0.067 | **0.161** | 0.001 | **0.135** | **0.111** | 1 |  |  |  |  |  |  |  |  |  |  |  |  |  |  |  |  |  |  |
|  | Anemia | 0.025 | **0.249** | 0.003 | 0.049 | 0.008 | 0.032 | 1 |  |  |  |  |  |  |  |  |  |  |  |  |  |  |  |  |  |
|  | WBC | **0.19** | **-0.143** | **0.117** | **0.118** | 0.065 | 0.037 | **-0.12** | 1 |  |  |  |  |  |  |  |  |  |  |  |  |  |  |  |  |
|  | RBC | **0.432** | **-0.282** | 0.034 | 0.052 | -0.008 | -0.039 | **-0.414** | **0.287** | 1 |  |  |  |  |  |  |  |  |  |  |  |  |  |  |  |
|  | HGB | **0.559** | **-0.25** | -0.011 | 0.051 | -0.027 | -0.04 | **-0.547** | **0.279** | **0.796** | 1 |  |  |  |  |  |  |  |  |  |  |  |  |  |  |
|  | PLT | -0.077 | **-0.163** | 0.077 | 0.011 | -0.08 | 0.015 | -0.041 | **0.238** | **0.103** | 0.025 | 1 |  |  |  |  |  |  |  |  |  |  |  |  |  |
|  | HCT | **0.558** | **-0.231** | -0.027 | 0.049 | -0.035 | -0.032 | **-0.54** | **0.279** | **0.831** | **0.946** | 0.039 | 1 |  |  |  |  |  |  |  |  |  |  |  |  |
|  | MCV | **0.244** | **0.13** | **-0.087** | 0.017 | -0.031 | 0.023 | -0.080 | -0.006 | **-0.178** | **0.2** | **-0.146** | **0.19** | 1 |  |  |  |  |  |  |  |  |  |  |  |
|  | MCH | **0.244** | **0.111** | -0.09 | 0.004 | -0.012 | -0.019 | **-0.113** | -0.02 | **-0.171** | **0.223** | **-0.142** | **0.167** | **0.867** | 1 |  |  |  |  |  |  |  |  |  |  |
|  | MCHC | 0.032 | -0.005 | -0.035 | 0.024 | **0.109** | -0.045 | **-0.176** | -0.057 | -0.071 | **0.139** | **-0.111** | 0 | **0.098** | **0.411** | 1 |  |  |  |  |  |  |  |  |  |
|  | FPG | 0.038 | -0.002 | 0.078 | 0.078 | **0.368** | 0.002 | **-0.084** | **0.125** | **0.094** | **0.098** | 0.066 | 0.063 | **-0.083** | -0.005 | **0.176** | 1 |  |  |  |  |  |  |  |  |
|  | BUN | **0.211** | 0.065 | 0.07 | 0.06 | **0.107** | 0 | **0.085** | 0.077 | 0.04 | 0.079 | -0.033 | 0.07 | 0.062 | 0.044 | 0.028 | **0.119** | 1 |  |  |  |  |  |  |  |
|  | CREA | **0.608** | 0.06 | 0.044 | **0.155** | -0.001 | -0.082 | **0.131** | 0.081 | **0.274** | **0.35** | **-0.099** | **0.366** | **0.21** | **0.164** | -0.023 | -0.029 | **0.320** | 1 |  |  |  |  |  |  |
|  | UA | **0.419** | 0.01 | 0.058 | **0.259** | 0.045 | -0.014 | 0.001 | **0.165** | **0.298** | **0.3** | -0.057 | **0.297** | 0.027 | 0.025 | 0.014 | 0.026 | **0.15** | **0.435** | 1 |  |  |  |  |  |
|  | TC | **-0.105** | **-0.144** | -0.003 | -0.056 | 0.006 | -0.057 | **-0.234** | **0.101** | **0.219** | **0.176** | **0.189** | **0.163** | -0.071 | -0.083 | 0.015 | **0.144** | 0.032 | -0.074 | 0.02 | 1 |  |  |  |  |
|  | TG | 0.064 | -0.041 | 0.06 | **0.183** | **0.193** | -0.031 | **-0.154** | **0.163** | **0.22** | **0.195** | 0.054 | **0.184** | -0.063 | 0.006 | **0.144** | **0.103** | -0.025 | 0.053 | **0.276** | **0.214** | 1 |  |  |  |
|  | HDL-C | **-0.331** | -0.017 | -0.11 | -**0.082** | **-0.111** | 0.022 | -0.078 | **-0.166** | **-0.108** | **-0.133** | 0.052 | **-0.14** | 0.001 | -0.036 | -0.049 | -0.059 | 0.023 | **-0.185** | **-0.279** | **0.195** | **-0.392** | 1 |  |  |
|  | LDL-C | -0.001 | **-0.151** | 0.016 | -0.058 | -0.032 | -0.051 | **-0.216** | 0.079 | **0.269** | **0.238** | **0.153** | **0.231** | -0.069 | **-0.085** | -0.01 | **0.142** | 0.013 | -0.033 | 0.012 | **0.807** | **0.097** | 0.066 | 1 |  |
|  |  | Male | Age | CAA | Hypertension | T2DM | CAD | Anemia | WBC | RBC | HGB | PLT | HCT | MCV | MCH | MCHC | FPG | BUN | CREA | UA | TC | TG | HDL-C | LDL-C |  |
